# Peptic Ulcer Disease Associated with Central Obesity

**DOI:** 10.3390/jpm12121968

**Published:** 2022-11-28

**Authors:** Song-Seng Loke, Wen-Cheng Li

**Affiliations:** 1 Division of Geriatric Medicine, Department of Family Medicine, Kaohsiung Chang Gung Memorial Hospital, Chang Gung University College of Medicine, 123, Dapi Road, Niaosong District, Kaohsiung 80708, Taiwan; 2 Department of Family Medicine, Chang Gung Memorial Hospital, Linkou Branch, College of Medicine, Chang-Gung University, Taoyuan 32023, Taiwan

**Keywords:** peptic ulcer disease, metabolic syndrome, central obesity, health examination

## Abstract

This retrospective cross-sectional study aimed to evaluate associations between peptic ulcer disease (PUD), bone mineral density, and metabolic syndrome (MetS) and its components in healthy populations. Data were collected from the health examination database of a tertiary medical center in southern Taiwan from January 2015 to December 2016. Subjects who had undergone metabolic factors assessment, upper gastrointestinal endoscopy, and dual energy X-ray absorptiometry scans were enrolled. In total, 5102 subjects were included, with mean age 52.4 ± 12.0 years. Among them, 1332 (26.1%) had PUD. Multivariate logistic regression analysis showed that age (OR 1.03, *p* < 0.001), male (OR 1.89, *p* < 0.001), diabetes (OR 1.23, *p* = 0.004), BMI (OR 1.03, *p* = 0.001), and GOT (OR 1, *p* = 0.003) are risk factors for PUD. Regarding MetS parameters, larger waist circumference (OR 1.26, *p* = 0.001) is associated with PUD, and high triglycerides (OR 1.20, 95% CI 1.01–1.43) is associated with gastric ulcer, while low HDL (OR 1.31, 95% CI 1.07–1.59) and osteoporosis (OR 1.44, 95% CI 1.08–1.91) are associated with duodenal ulcer. In conclusion, central obesity is associated with PUD in a middle-aged healthy population. Subjects with high triglycerides are prone to gastric ulcers, and those with osteoporosis and low HDL are prone to duodenal ulcers.

## 1. Introduction

Peptic ulcer disease (PUD) is a common upper gastrointestinal disease. It has been found to be caused by *Helicobacter pylori* (*H. pylori*) infection, the overuse of nonsteroidal anti-inflammatory drugs or aspirin, smoking and alcohol consumption [1,2]. If not well treated, PUD may cause gastrointestinal bleeding, obstruction and perforation, which increases the mortality risk and socioeconomic burden [3]. 

Osteoporosis is a chronic skeletal disease characterized by reduced bone mass. It is a prevalent public health problem in the elderly population, with high fracture risk leading to high morbidity and mortality [4]. Metabolic syndrome (MetS) is a complex multifactorial disorder comprising central obesity, hyperglycemia, hypertriglyceridemia, high blood pressure, and a low concentration of high-density lipoprotein cholesterol (HDL-C). Besides the association with diabetes mellitus and cardiovascular disease, MetS may also be associated with bone metabolism [5,6] and central obesity [7]. 

*H. pylori* infection causes a chronic inflammation in the gastric and duodenal mucosa, leading to PUD. This inflammation may not be confined to the digestive tract but also may result in extra-digestive conditions such as osteoporosis [8]. However, the relationship is controversial. An association between *H. pylori* infection and osteoporosis was reported in a systematic review and meta-analysis by Wang et al. [9], but Kakehasi et al. [10] did not find such an association. However, *H. pylori* infection is associated with MetS [11,12,13]. Although PUD, MetS, and osteoporosis are prevalent in the general population, the relationship between PUD, MetS components, and osteoporosis have not been fully studied. This study aimed to investigate the associations between PUD, MetS and its components, and low bone mineral density in a middle-aged healthy population.

## 2. Materials and Methods

### 2.1. Study Design and Subjects

This retrospective cross-sectional study was performed using data extracted from the database of the Health Management and Evaluation Center of a tertiary medical center located in southern Taiwan from populations undergoing health examinations between January 2015 and December 2016. The center offers a variety of medical tests and procedures as part of routine physical examinations. Most of the health examinees were undergoing a self-paid physical check-up; others were employees coming for their regular medical check-up. Among 10219 subjects who underwent routine medical check-up at the health examination center, most were free of symptoms. Inclusion criteria were: (1) assessed for metabolic factors; (2) underwent upper gastrointestinal endoscopy and dual energy X-ray absorptiometry scan (DEXA). Exclusion criteria were: (1) did not undergo upper gastrointestinal endoscopy or DEXA; (2) incomplete data; (3) previous or current gastric cancer. Finally, a total of 5102 subjects were included for data analysis. The study protocol was approved by the Chang Gung Medical Foundation Institutional Review Board (IRB No.: 202200895B0) before the study. Signed informed consent of subjects was waived because of the anonymous retrospective nature of the study.

### 2.2. Measurement of Anthropometric Parameters and Bone Mineral Density

Body weight and height were measured using the same testing equipment (HW-3030, Super-View Medical, Hualien, Taiwan) at the same time with accuracy of 0.1 kg and 1 mm, respectively, as subjects stood erect and were barefoot and wearing light clothing. Body mass index (BMI) was then calculated as weight in kilograms divided by height in meters squared (kg/m^2^). Waist circumference (WC) was measured in centimeters at the mid-level between the iliac crest and the lower border of the 12th rib while subjects stood with feet 25–30 cm apart. BMD values were measured in g/cm^2^ by DEXA (Lunar Prodigy Advance; GE Healthcare, Madison, WI, USA) at the lumbar spine, total femoral (total hip), and femoral neck.

### 2.3. Definition of Metabolic Syndrome and Low Bone Mineral Density

In this study, MetS was defined according to the modified National Cholesterol Education Program Adult Treatment Panel III (NCEP ATP III) for Asian populations [14]. The modified NCEP ATP III criteria suggested that the cut-off points for WC of Asian populations should use the cut-off of 90 cm in men and 80 cm in women. MetS was diagnosed when at least three of the following five components were found: (1) WC ≥ 90 cm for men and ≥80 cm for women; (2) high blood pressure (systolic blood pressure ≥130 mm Hg and/or diastolic pressure ≥85 mm Hg, under treatment, or already diagnosed with hypertension); (3) high serum triglyceride (≥150 mg/dL); (4) decreased HDL-C (<40 mg/dL for males and <50 mg/dL for females); and (5) high fasting glucose (FG) ≥ 100 mg/dL, under treatment, or previously diagnosed with diabetes mellitus. 

The diagnosis of osteoporosis was defined according to the World Health Organization (WHO) definition. The T-score was calculated automatically; the lowest value was chosen for the diagnosis of osteoporosis. Osteoporosis was defined as T-score ≦ –2.5; osteopenia as −2.5 < T-score < −1, normal as ≧−1. A definition of low BMD included both osteopenia and osteoporosis.

### 2.4. Statistical Analyses

Continuous data are presented as mean ± standard deviation (SD) and analyzed by Student’s t test. Categorical data are presented as *n* (%) and analyzed by the chi-square test. Univariate and multivariate logistic regression was applied to calculate adjusted odds ratios (aOR) and 95% confidence intervals (CI) to evaluate associations between outcomes and covariates. Covariates with *p*-value < 0.05 were identified as potential risk factors for outcomes based on the stepwise selection method using logistic regression. The covariates and MetS were entered into model 1, and components of MetS were instead entered as MetS into model 2. Selected covariates were placed into multivariate logistic regression to assess the associations between variables and outcomes, including PUD, gastric ulcer, and duodenal ulcer. All *p* values were two-sided and *p*-value < 0.05 was established as statistical significance. All statistical analyses were performed using the statistical software package SAS software version 9.4 (SAS Institute Inc., Cary, NC, USA).

## 3. Results

### 3.1. Prevalence of Peptic Ulcer Disease and Metabolic Syndrome

A total of 5102 subjects were included, with 2159 (42.3%) females and 2943 (57.7%) males. The mean age was 52.4 ± 12.0 years. The prevalence of asymptomatic PUD was 26.1%, including 880 subjects with gastric ulcer and 601 subjects with duodenal ulcer. Additionally, 31.8% subjects were diagnosed with MetS.

### 3.2. Differences between Subjects with Peptic Ulcer Disease and Non-Peptic Ulcer Disease

Table 1 shows the baseline characteristics between subjects with and without PUD. Subjects with PUD were significantly older (55.9 ± 10.9 vs. 51.2 ± 12.1 years, *p* < 0.001), with significantly higher BMI (24.7 ± 3.7 vs. 23.9 ± 3.8 kg/m, *p* < 0.001), WC (84.5 ±10.7 vs. 81.3 ±10.8 cm, *p* < 0.001), systolic blood pressure (SBP) (128.2 ± 19.2 vs. 123.8 ± 18.7 mmHg, *p* < 0.001), diastolic blood pressure (DBP) (84.8 ± 10.9 vs. 82.6 ± 11.0 mmHg, *p* < 0.001), fasting glucose (107.1 ± 30.2 vs. 101.4 ± 24.0 mg/dL, *p* < 0.001), triglycerides (129.2 ± 38.6 vs. 120.8 ± 39.5 mg/dL, *p* < 0.001), low-density lipoprotein cholesterol (LDL-C) (128.8 ± 34.1 vs. 126.3 ± 35.3 mg/dL, *p* = 0.025), uric acid (6.6 ± 1.6 vs. 6.3 ± 1.6 mg/dL, *p* < 0.001), GOT (28.3 ± 20.3 vs. 25.7 ± 14.4 U/L, *p* < 0.001) and GPT (32.9 ± 32.7 vs. 29.1 ± 22.6 U/L, *p* < 0.001) compared to those in subjects without PUD. The levels of HDL-C (49.7 ± 13.2 vs. 52.0 ± 13.8 mg/dL, *p* < 0.001) and eGFR (85.9 ± 20.4 vs. 90.9 ± 20.0 mL/min/1.73 m^2^, *p* < 0.001) in subjects with PUD were significantly lower than those in subjects without PUD.

### 3.3. Univariate and Multivariate Logistic Regression Analyses of Variables Associated with Peptic Ulcer Disease

Results from univariate and multivariate logistic regression analyses are shown in Table 2. Most covariates were associated with higher risk for PUD, except reflux esophagitis and low HDL-C. Model 1 (covariates + MetS) showed that age, sex, BMI, GOT, diabetes, and reflux esophagitis were significantly associated with PUD. Older patients (aOR = 1.03; 95%: 1.03–1.04), male (aOR = 1.80; 95%: 1.56–2.07) or patients with higher BMI (aOR = 1.03; 95%: 1.01–1.05), higher GOT (aOR = 1.00; 95%: 1.00–1.01), diabetes (aOR = 1.23; 95%: 1.02–1.49) had higher risk for PUD. However, reflux esophagitis demonstrated a protective effect for PUD (aOR = 0.81; 95%: 0.70–0.94). Model 2 had similar results, but large WC was a risk factor (aOR = 1.26; 95%: 1.10–1.44), while BMI was not.

Table 3 shows that most covariates had significant effects on gastric ulcer apart from hyperlipidemia, reflux esophagitis, and low HDL-C. According to the results from model 1 (covariates + MetS), the covariates increased the risk of gastric ulcer, including age (aOR = 1.03; 95%: 1.02–1.04), male (aOR = 1.46; 95%: 1.24–1.71), BMI (aOR = 1.06; 95%: 1.04–1.08), GOT (aOR = 1.004; 95%: 1.00–1.01), and diabetes (aOR = 1.32; 95%: 1.07–1.63). However, reflux esophagitis decreased the risk of developing gastric ulcer (aOR = 0.82; 95%: 0.69–0.97). High triglycerides (aOR = 1.20; 95%: 1.01–1.43) were selected as a new risk factor in model 2, and the results of remaining variables were as in model 1.

Table 4 lists the covariates significantly associated with duodenal ulcer, including age, sex, eGFR, GOT, GPT, diabetes, osteoporosis, high BP, high fasting glucose in univariate logistic regression. In multivariate logistic regression analysis, age (aOR = 1.03; 95%: 1.02–1.04), male (aOR = 2.20; 95%: 1.81–2.67), and BMD (osteopenia vs. normal; aOR = 1.42; 95%: 1.07–1.88) significantly increased the risk of duodenal ulcer in model 1. Furthermore, compared with model 1, more selected factors were found in model 2, including low HDL-C (aOR = 1.31; 95%: 1.07–1.59) and high triglycerides (aOR = 0.67; 95%: 0.55–0.85).

## 4. Discussion

The prevalence of asymptomatic PUD and MetS are increasing in Taiwan. The prevalence of asymptomatic PUD was 9.4% in 2008 [15] and age-standardized prevalence of MetS was 15.7% by the modified ATP III criteria in 2002 [16]. In the present study, the prevalence of PUD and MetS was 26.1% and 31.9%, respectively. Results of the present study show that the dominant risk factors for PUD in a healthy middle-aged population are age, sex, GOT, diabetes, and large WC. MetS is not associated with PUD, but gastric ulcer is associated with a high TG level, while duodenal ulcer is associated with a low HDL level and osteoporosis. 

The present study shows that BMI and large WC are associated with PUD. Previous studies have proposed that abdominal obesity implicates digestive system diseases because the altered secretion of adipocytokines from accumulated visceral fat is associated with various pathophysiological conditions, including insulin resistance and inflammation. This inflammation may further cause gastrointestinal mucosal injury, leading to PUD [17,18]. Nevertheless, the relationship between PUD and obesity, including total obesity and central obesity, remains controversial [7,15,19,20,21]. Some studies have reported obesity as a risk factor for PUD [7,15,20,21], but Tsai et al. [19] did not observe a significant association between BMI and PUD. The underlying mechanism that prompts obesity to increase the risk of asymptomatic PUD is unclear. Several possible explanations have been suggested. In obese subjects, increased intra-abdominal pressure may cause more acid secretion [22]. Wisén et al. [23] used the gastric acid secretion test after modified sham feeding to show that obese patients had higher gastric acid secretion than non-obese patients. Adipose tissue is an endocrine organ involved in the development and occurrence of inflammation by secreting adipokines [24,25]. Adipose tissue and inflammatory cells may promote inflammation and insulin resistance, resulting in the expression of adhesion molecules and pro-inflammatory cytokines [26]. These factors together induce chronic low-grade inflammation of the body and induce PUD.

The present study has shown that MetS is not associated with PUD, but its components are. Triglycerides are positively associated with gastric ulcer but negatively associated with duodenal ulcer, while low HDL is associated with duodenal ulcer. The relationship between serum lipid levels and PUD varies in different populations. Kim et al. [27] showed that triglycerides are associated with PUD in men but not in women. In addition, *H. pylori* infection significantly affects serum lipid profiles. Studies have reported that *H. pylori* infection is positively associated with TG and negatively associated with HDL-C [11,28]. Further study is needed to determine why triglycerides display conflicting associations between gastric and duodenal ulcers.

Results of the present study have shown that duodenal ulcers are associated with osteoporosis, which is probably related to reduced calcium absorption in the inflamed duodenal mucosa. Several studies have also shown an association between PUD and osteoporosis [29,30,31], but a systematic review and cumulative analysis found that only men had a positive association between PUD and osteoporosis, while women with PUD did not have a higher risk of osteoporosis when compared with the general population [32]. 

Diabetes mellitus is associated with PUD, especially gastric ulcer. Previous studies have reported a high prevalence of PUD in patients with diabetes mellitus [33,34,35]. A possible explanation is the significantly higher prevalence of *H. pylori* infection in patients with type II diabetes [36]. In addition, diabetic angiopathy and the use of antiplatelet medications in diabetic patients may impair the integrity of the gastric mucosa, causing ulcer formation [37].

Besides the factors stated above, gut microbiota also plays important roles in many diseases including obesity and metabolic syndromes [38]. It was reported that intestinal microbiota controlled Th17 cells which regulated innate immune response and lipid absorption across intestinal epithelium [39,40]. Intestinal microbiota can also degrade indigestible polysaccharides and secrete short chain fatty acids (SCFA) which can be transported elsewhere and affect cellular function [41]. Insufficient amount of SCFA weakens the tight junction and makes the intestinal epithelial barrier leaky [42,43]. This facilitates the infection of *H. pylori* and the progressing of inflammation bowel diseases [38,44].

The present study analyzed the risk factors for PUD in middle-aged population. Keeping regular hours, ration size, and meal schedule are important for high-risk populations. To avoid PUD, good control of BMI, waist circumference, GOT, and triglycerides level is recommended for male subjects, especially for those with old age or diabetes. For male subjects with osteoporosis, good control of HDL level is recommended to avoid duodenal ulcer.

The present study has several limitations. First, the data were extracted from an administrative computer database that did not include medication and lifestyle factors such as aspirin use, smoking, and alcoholic consumption that may induce PUD formation and therefore, these factors could not be adjusted in the statistical models. Second, the retrospective cross-sectional study design did not allow causal relationships to be inferred. Third, the study subjects were from a single health-promotion center in southern Taiwan and study results may not be representative of the general population or other ethnic populations. Fourth, the data from 2015–2016 were used because of the largest population size for analysis. Hence, they may not completely reflect the current associations.

## 5. Conclusions

Central obesity is associated with PUD in a general middle-aged population. High triglycerides are associated with gastric ulcer, while low HDL-C and osteoporosis are associated with duodenal ulcer. Further prospective cohort studies are warranted to clarify the cause-and-effect relationships.

## Figures and Tables

**Table 1:** Differences in baseline characteristics between peptic ulcer disease and non-peptic ulcer disease groups.

	All (*n* = 5102)	Peptic Ulcer Disease (*n* = 1332)	Non-Peptic Ulcer Disease (*n* = 3770)	*p* Value
**Demography & laboratory data**				
Age, years	52.4 ± 12.0	55.9 ± 10.9	51.2 ± 12.1	<0.001 *.
Sex				<0.001 *
Female, *n* (%)	2159 (42.3)	413 (31.0)	1746 (46.3)	
Male, *n* (%)	2943 (57.7)	919 (69.0)	2024 (53.7)	
BMI, kg/m^2^	24.2 ± 3.8	24.7 ± 3.7	23.9 ± 3.8	<0.001 *
WC, cm	82.1 ± 10.9	84.5 ±10.7	81.3 ±10.8	<0.001 *
BP, mmHg				
Systolic	124.9 ± 18.9	128.2 ± 19.2	123.8 ± 18.7	<0.001 *
Diastolic	83.2 ± 11.0	84.8 ± 10.9	82.6 ± 11.0	<0.001 *
Fasting glucose, mg/dL	102.9 ± 25.9	107.1 ± 30.2	101.4 ± 24.0	<0.001 *
Total cholesterol, mg/dL	205.2 ± 39.3	206.8 ± 38.6	204.7 ± 39.5	0.09
HDL-C, mg/dL	51.4 ± 13.7	49.7 ± 13.2	52.0 ± 13.8	<0.001 *
Triglycerides, mg/dL	123.0 ± 87.0	129.2 ± 88.4	120.8 ± 86.5	0.002 *
LDL-C, mg/dL	127.0 ± 35.0	128.8 ± 34.1	126.3 ± 35.3	0.025 *
Uric acid, mg/dL	6.4 ± 1.6	6.6 ± 1.6	6.3 ± 1.6	<0.001 *
eGFR, mL/min/1.73 m^2^ (missing value = 2)	89.6 ± 20.2	85.9 ± 20.4	90.9 ± 20.0	<0.001 *
GOT, U/L (missing value = 3)	26.4 ± 16.2	28.3 ± 20.3	25.7 ± 14.4	<0.001 *
GPT, U/L (missing value = 2)	30.1 ± 25.7	32.9 ± 32.7	29.1 ± 22.6	<0.001 *
**Comorbidities**				
Diabetes, *n* (%)	591 (11.6)	208 (15.6)	383 (10.2)	<0.001 *
Hypertension, *n* (%)	1115 (21.9)	359 (27.0)	756 (20.1)	<0.001 *
Hyperlipidemia, *n* (%)	214 (4.2)	74 (5.6)	140 (3.7)	0.004 *
Reflux esophagitis, *n* (%)	1452 (28.5)	374 (28.1)	1078 (28.6)	0.72
BMD, *n* (%)				
Normal	2553 (50.0)	589 (44.2)	1964 (52.1)	<0.001 *
Osteopenia	1903 (37.3)	539 (40.5)	1364 (36.2)	
Osteoporosis	646 (12.7)	204 (15.3)	442 (11.7)	
MetS, *n* (%)	1620 (31.8)	502 (37.7)	1118 (29.7)	<0.001 *

* *p* < 0.05. BMI: Body mass index, BMD: Bone mineral density, BP: Blood pressure, HDL-C: High-density lipoprotein cholesterol, LDL-C: Low density lipoprotein cholesterol, MetS: Metabolic syndrome.

**Table 2:** Risk factors for peptic ulcer disease.

Variables	Univariate		Multivariate			
			Model 1		Model 2	
	OR (95% CI)	*p* Value	aOR (95% CI)	*p* Value	aOR (95% CI)	*p* Value
**Demography & laboratory data**						
Age	1.04 (1.03–1.04)	<0.001 *	1.03 (1.03–1.04)	<0.001 *	1.03 (1.03–1.04)	<0.001 *
Sex (Male vs. Female)	1.92 (1.68–2.19)	<0.001 *	1.80 (1.56–2.07)	<0.001 *	1.89 (1.65–2.17)	<0.001 *
BMI	1.06 (1.04–1.08)	<0.001 *	1.03 (1.01–1.05)	0.001 *		
Uric acid	1.00 (1.00–1.00)	0.025 *				
eGFR	0.99 (0.98–0.99)	<0.001 *				
GOT	1.01 (1.01–1.01)	<0.001 *	1.004 (1.00–1.01)	0.035 *	1.00 (1.00–1.01)	0.003 *
GPT	1.01 (1.00–1.01)	<0.001 *				
**Comorbidities (vs. No/Normal)**						
Diabetes	1.64 (1.37–1.96)	<0.001 *	1.23 (1.02–1.49)	0.033 *	1.23 (1.01–1.48)	0.004 *
Hypertension	1.47 (1.27–1.70)	<0.001 *				
Hyperlipidemia	1.53 (1.14–2.04)	0.004 *				
Reflux esophagitis, *n* (%)	0.98 (0.85–1.12)	0.720	0.81 (0.70–0.94)	0.004 *	0.81 (0.70–0.94)	0.005 *
BMD (vs. Normal)						
Osteopenia	1.31 (1.15–1.51)	<0.001 *				
Osteoporosis	1.54 (1.27–1.86)	<0.001 *				
**MetS and parameters (vs. No/Normal)**						
MetS	1.44 (1.26–1.64)	<0.001 *			-------	-------
Large WC	1.42 (1.25–1.62)	<0.001 *	-------	-------	1.26 (1.10–1.44)	0.001 *
High BP	1.43 (1.26–1.62)	<0.001 *	-------	-------		
High Fasting glucose	1.54 (1.36–1.75)	<0.001 *	-------	-------		
High Triglycerides	1.22 (1.06–1.40)	0.006 *	-------	-------		
Low HDL-C	1.10 (0.96–1.26)	0.183	-------	-------		

Model 1: covariates + MetS using stepwise selection to select risk factors. Model 2: covariates + MetS parameters using stepwise selection to select risk factors. *: Indicates a significant difference, *p* < 0.05. BMI: Body mass index, BMD: Bone mineral density, BP: Blood pressure, HDL-C: High-density lipoprotein cholesterol, LDL-C: Low density lipoprotein cholesterol, MetS: Metabolic syndrome, WC: Waist circumference.

**Table 3:** Risk factors for gastric ulcer disease.

Variables	Univariate		Multivariate			
			Model 1		Model 2	
	OR (95% CI)	*p* Value	aOR (95% CI)	*p* Value	aOR (95% CI)	*p* Value
**Demography & laboratory data**						
Age	1.03 (1.03–1.04)	<0.001 *	1.03 (1.02–1.04)	<0.001 *	1.03 (1.02–1.04)	<0.001 *
Sex (Male vs. Female)	1.66 (1.420–1.93)	<0.001 *	1.46 (1.24–1.71)	<0.001 *	1.43 (1.22–1.68)	<0.001 *
BMI	1.08 (1.06–1.10)	<0.001 *	1.06 (1.04–1.08)	<0.001 *	1.05 (1.03–1.08)	<0.001 *
Uric acid	1.00 (1.00–1.00)	0.027 *				
eGFR	0.99 (0.98–0.99)	<0.001 *				
GOT	1.01 (1.01–1.01)	<0.001 *	1.00 (1.00–1.01)	0.034 *	1.00 (1.00–1.01)	0.045 *
GPT	1.01 (1.00–1.01)	<0.001 *				
**Comorbidities (vs. No/Normal)**						
Diabetes	1.74 (1.43–2.13)	<0.001 *	1.32 (1.07–1.63)	0.010 *	1.31 (1.06–1.62)	0.012 *
Hypertension	1.57 (1.34–1.85)	<0.001 *				
Hyperlipidemia	1.37 (0.98–1.91)	0.063				
Reflux esophagitis, *n* (%)	0.97 (0.83–1.14)	0.716	0.82 (0.69–0.97)	0.018 *	0.81 (0.69–0.96)	0.014 *
BMD (vs. Normal)						
Osteopenia	1.37 (1.17–1.60)	<0.001 *				
Osteoporosis	1.25 (0.99–1.57)	0.057				
**MetS and parameter (vs. No/Normal)**						
MetS	1.60 (1.38–1.86)	<0.001 *			-------	-------
Large WC	1.56 (1.35–1.81)	<0.001 *	-------	-------		
High BP	1.44 (1.25–1.67)	<0.001 *	-------	-------		
High Fasting glucose	1.65 (1.43–1.91)	<0.001 *	-------	-------		
High Triglycerides	1.45 (1.24–1.70)	<0.001 *	-------	-------	1.20 (1.01–1.43)	0.034 *
Low HDL-C	1.14 (0.97–1.33)	0.103	-------	-------		

Model 1: covariates + MetS using stepwise selection to select risk factors. Model 2: covariates + MetS parameters using stepwise selection to select risk factors. *: Indicates a significant difference, *p* < 0.05 BMI: Body mass index, BMD: Bone mineral density, BP: Blood pressure, HDL-C: High-density lipoprotein cholesterol, LDL-C: Low density lipoprotein cholesterol, MetS: Metabolic syndrome, WC: Waist circumference.

**Table 4:** Risk factors for duodenal ulcer disease.

Variables	Univariate		Multivariate			
			Model 1		Model 2	
	OR (95% CI)	*p* Value	aOR (95% CI)	*p* value	aOR (95% CI)	*p* Value
**Demography & laboratory data**						
Age	1.03 (1.03–1.04)	<0.001 *	1.03 (1.02–1.04)	<0.001 *	1.03 (1.02–1.04)	<0.001 *
Sex (Male vs. Female)	2.12 (1.76–2.56)	<0.001 *	2.20 (1.81–2.67)	<0.001 *	2.35 (1.92–2.86)	<0.001 *
BMI	1.02 (0.96–1.04)	0.134				
Uric acid	1.00 (0.99–1.00)	0.539				
eGFR	0.99 (0.98–0.99)	<0.001 *				
GOT	1.01 (1.00–1.01)	0.010 *				
GPT	1.00 (1.00–1.01)	0.004 *				
**Comorbidities (vs. No/Normal)**						
Diabetes	1.49 (1.18–1.90)	0.001 *				
Hypertension	1.21 (0.99–1.47)	0.064				
Hyperlipidemia	1.34 (0.91–1.96)	0.142				
Reflux esophagitis, *n* (%)	1.76 (0.86–1.30)	0.444				
BMD (vs. Normal)						
Osteopenia	1.19 (0.99–1.44)	0.065	0.88 (0.71–1.09)	0.233	0.88 (0.71–1.09)	0.254
Osteoporosis	1.89 (1.49–2.40)	<0.001 *	1.42 (1.07–1.88)	0.015 *	1.44 (1.08–1.91)	0.012 *
**MetS and parameter (vs. No/Normal)**						
MetS	1.13 (0.94–1.35)	0.186			-------	-------
Large WC	1.18 (0.99–1.41)	0.066	-------	-------		
High BP	1.27 (1.07–1.51)	0.006 *	-------	-------		
High Fasting glucose	1.33 (1.12–1.58)	0.001 *	-------	-------		
High Triglycerides	0.85 (0.70–1.04)	0.118	-------	-------	0.67 (0.55–0.85)	0.001 *
Low HDL-C	1.14 (0.95–1.37)	0.167	-------	-------	1.31 (1.07–1.59)	0.007 *

Model 1: covariates + MetS using stepwise selection to select risk factors. Model 2: covariates + MetS parameters using stepwise selection to select risk factors. *: Indicates a significant difference, *p* < 0.05 BMI: Body mass index, BMD: Bone mineral density, BP: Blood pressure, HDL-C: High-density lipoprotein cholesterol, LDL-C: Low density lipoprotein cholesterol, MetS: Metabolic syndrome, WC: Waist circumference.

## Data Availability

The datasets generated and/or analyzed during the present study are available from the corresponding author on reasonable request.
